# A ‘novel’ model for integrating Sport and Exercise Medicine (SEM) and Musculoskeletal (MSK) management into primary care in the UK

**DOI:** 10.1136/bmjsem-2015-000027

**Published:** 2015-09-15

**Authors:** Neil Heron

**Affiliations:** 1 Department of General Practice and Primary Care, Queen's University Belfast, Belfast, Northern Ireland; 2 Centre for Public Health, Queen's University Belfast, Belfast, Northern Ireland; 3 UKCRC Centre of Excellence for Public Health (NI), Queen's University Belfast, Belfast, Northern Ireland

**Keywords:** Health promotion, Intervention efficacy, Medicine, Physical activity promotion in primary care, Sporting injuries

## Abstract

**Background:**

Musculoskeletal (MSK) symptoms are common within primary care but some general practitioners (GPs)/family physicians do not feel comfortable managing these symptoms, preferring to refer onwards. We aimed to establish a reproducible GP-staffed MSK and sport and exercise medicine (SEM) clinic within primary care, in keeping with recent policy changes within the UK health system.

**Methods:**

A monthly MSK and SEM clinic was held within a Belfast GP practice, staffed by 1 GP with a specialist interest in MSK/SEM conditions, and its performance was reviewed over two 3-month periods. Parameters audited included diagnoses, patient satisfaction and secondary care referral rates.

**Results:**

83 patients, 36 males and 47 females, were reviewed in the clinic and the main presenting joint was the shoulder. Patient self-reported satisfaction with the service was high. Comparing referral rates between August and October 2013 and the same period in 2014, overall referrals from the practice were reduced by 147, orthopaedic and rheumatology referrals were reduced by 2 and 3, while physiotherapy and X-ray referrals were reduced by 47 and 90, respectively. Comparing the referral rates between January and March 2014 and the same period in 2015, overall outpatient referrals were reduced by 152, orthopaedic and rheumatology referrals were reduced by 9 and 4, while physiotherapy and X-ray referrals were reduced by 41 and 3, respectively.

**Discussion:**

We present a novel, reproducible service model for managing MSK/SEM symptoms in primary care which could be commissioned by local groups. This model can make sound economic sense and deliver high patient satisfaction within primary care, reducing waiting times and the secondary care referral burden.

**What we already know**

- Musculoskeletal/sport medicine injuries make up approximately 20% of the general practitioner (GP) workload.
- GPs often do not feel comfortable in managing musculoskeletal/sport medicine injuries, and therefore a high referral burden to secondary care often results.
- Within the UK health system, there is now an emphasis on shifting patient management from secondary and tertiary care into the community, with subsequent management pressures for community health workers, including GPs. New service models are needed to manage this demand.

## Background

With approximately 20% of general practitioners’ (GPs)/family physicians’ workload being related to musculoskeletal (MSK) conditions,^1–3^ GPs and primary care staff need to have access to appropriate clinics for investigating and managing MSK conditions. Moreover, primary care staff commonly report low confidence levels in managing common MSK conditions,^3^ further increasing the need for specialist MSK and, a related discipline, sport and exercise medicine (SEM) knowledge within GP.

The current health system within the UK dictates that patients with MSK/SEM symptoms are initially reviewed by the GP and then referred on to secondary care when further investigation and management is required. The secondary care options include an appointment in hospital with an orthopaedic specialist or review within orthopaedic Integrated Clinical Assessment and Treatment units (ICATs), typically within a community-based healthcare centre. However, waiting times for these specialties are long and waiting times for elective care are a common source of patient dissatisfaction within healthcare systems.^4^

**What this study adds**

- This study extends the analysis of the implementation of a unique musculoskeletal/sport and exercise medicine into routine GP to 6 months and builds on our previously published work.^6^
- A musculoskeletal and sport and exercise medicine clinic, run by a GP with a specialist interest in these conditions, can be successfully integrated into a local GP clinic.
- This service model has the potential to reduce referrals to secondary care, produce high patient satisfaction and make sound economical sense with significant cost savings.
- More wide scale studies are required to ensure that these results can be replicated throughout the UK healthcare system and beyond.

If GPs had appropriate specialist knowledge in this area, then the patients’ management could largely be within the local GP practice with secondary care input only for a select few patients not able to be managed appropriately by the GP, consequently decreasing the waiting times for elective care in hospitals. This latter approach would be supported by the Transforming Your Care policy in Northern Ireland (NI), with a shift in emphasis now from hospital-based to community-based management options.^5^ The ICAT service model has illustrated that GPs with a specialist interest can manage common MSK symptoms competently, but this project is developing this idea further by demonstrating that GPs with a specialist interest can manage these patients within their own practices.

## Aim

To develop a reproducible GP-staffed MSK and SEM clinic based within primary care that is economically sound and sustainable within the current National Health Service (NHS) climate, providing high patient satisfaction.

## Methods

This quality improvement project was conducted in a Belfast GP practice of approximately 9000 patients and five GP partners. The study methodology and provisional results have been previously reported,^6^ but the current study develops the initial paper^6^ further by extending the analysis to 6 months.

The GP practice uses Egton Medical Information Systems (EMIS) electronic patient records and is based approximately two miles from Belfast city centre. The practice introduced an MSK and SEM clinic staffed by one GP with a specialist interest in MSK and SEM conditions, with appropriate postgraduate qualifications in these areas, and its performance was reviewed over two 3-month time periods between August and October 2014 and then again between January and March 2015. A monthly 4 h clinic was held over these time periods and appointment times were approximately 20 min. All primary care staff within the practice could refer to the clinic any patient with MSK and/or SEM presentations, on whom they wanted some specialist input. Management options included injection therapy, exercise prescription and onward referrals to appropriate colleagues, for example, physiotherapy. No ethical approval was required for this study as it was an audit of a new service within routine GP.

The practice's performance during the running of the clinic was compared with the same time periods in 2013 and 2014 to control for other variables except for the introduction of the GP-based MSK and SEM clinic. The variables which were monitored included cases seen in the clinic, waiting times, treatments given and onward referral to colleagues from the clinic.

Equipment which was available to the GP within the GP-based MSK and SEM clinic included a clinical room with a computer to allow access to the patients’ GP clinical records, a couch and lighting. There were also facilities to undertake joint injections under appropriate sterile conditions. Within the primary care clinic, there was access to laboratory blood tests (to exclude an inflammatory condition) and imaging, including open access radiology for plain films. If needed, patients could be referred for input to orthopaedic ICATs and hospital specialties, including rheumatology and orthopaedics, using the typical referral methods.

Ten patients were randomly selected to complete a patient satisfaction questionnaire (see online supplementary appendix 1). The patients were approached by the practice administration staff to complete the questionnaire via a paper copy after they had finished their clinic visit. This questionnaire was developed from a recognised patient satisfaction questionnaire for assessing GP services,^7^ with input from the Patient Experience Group at the Royal Group of Hospitals, Belfast.

## Results

### GP-staffed MSK and SEM clinic performance

#### August to October 2014

Thirty-five patients were seen in the GP-staffed MSK and SEM clinic, 14 males and 21 females, between August and October 2014. The age range of patients seen was from 35 to 77 years. The patients were generally referred from the other GPs within the practice, but a minority of referrals were also sent from physiotherapy, podiatry and hospital colleagues. For example, one patient was referred to the clinic from rheumatology due to the long waiting times for joint injections within their department.

With regard to management in the GP-based clinic, as well as the steroid injection, the patients were also given advice regarding appropriate conservative management options, including analgesia, muscle stretches and strengthening exercises. The most common management option employed within the clinic was the steroid injection. A source of appropriate patient information used within the clinic was from Arthritis Research UK (ARUK), who provide information leaflets for patients on various MSK symptoms.^8^

The main joint presenting to the clinic was the shoulder, with the main pathology detected here being within the supraspinatus muscle (table 1).

**Table 1:** Main joints presenting in the musculoskeletal/sport and exercise medicine general practitioner clinic between August and October 2014

Joint with presenting symptom	Number of times presenting
Shoulder	13
Knee	6
Hip	5
Hand	4
Elbow	4
Foot	4

*Previously published (6).

#### January to March 2015

Forty-eight patients were seen in the GP-staffed MSK and SEM clinic within this period, 22 males and 26 females. The age range of patients seen was from 26 to 77 years. All patients were referred by a GP in the practice for further assessment of their symptoms. The main joint presenting to the clinic was again the shoulder, with the main clinical diagnosis detected here being supraspinatus bursitis and the primary management option being the local steroid injection (table 2).

**Table 2:** Main joints presenting to the musculoskeletal/sport and exercise medicine general practitioner clinic between January and March 2015

Joint with presenting symptom	Number of times presenting
Shoulder	18
Elbow	7
Knee	7
Foot	6
Hip	6
Hand/wrist	5

### Patient satisfaction questionnaire responses

Ten participants were randomly selected from the six clinics held to complete a patient satisfaction questionnaire (see online supplementary appendix 1). This included four males and six females of mean age 51 years (to the nearest year). Their responses are summarised in table 3. The patients generally felt that it was easy to get an appointment at the GP-based clinic, with the investigating doctor generally doing enough tests and patients having high levels of confidence in the treating GP. Communication at the clinic was clear, with high patient satisfaction with the services offered. Patients appeared happier to have their conditions managed within their GP surgery than compared with hospital or another community-based health facility and would have prefer to see future specialists within their GP clinic rather than without. There was also a space at the bottom of the questionnaire for free text comments and three different people left a comment, included in online supplementary appendix 2. The patients’ comments illustrated how highly they valued the service at the GP clinic.

**Table 3:** Patient satisfaction questionnaire responses

Question	Number of times selected				
	Strongly agree (1)	Agree (2)	Neither agree nor disagree (3)	Disagree (4)	Strongly disagree (5)
1. Getting an appointment for the GP-based orthopaedic clinic at a convenient time was easy?	9	1			
2. The doctor did enough tests to find out what was wrong with me?	8	1	1		
3. I have absolute faith and confidence in the doctor at the GP-based orthopaedic clinic?	9	1			
4. The doctor at the GP-based orthopaedic clinic did not tell me enough about the treatment?				1	9
5. The doctor at the GP-based orthopaedic clinic fully explained how the illness and treatment would affect my future health?	6	4			
6. Appointments are easy to make whenever I need them at the GP-based orthopaedic clinic?	6	4			
7. I felt perfectly satisfied with the way I was treated at the surgery when I attended the GP-based orthopaedic clinic?	9	1			
8. The doctor showed a genuine interest in my problems at the GP-based orthopaedic clinic?	9	1			
9. The doctor always puts me at ease at the GP-based orthopaedic clinic?	9	1			
10. My general experience at the GP-based orthopaedic clinic was very good?	9	1			
11. My experience at the GP-based orthopaedic clinic was generally better than if I had been referred to hospital for an orthopaedic outpatient review?	9	1			

*Previously published (6).

GP, general practitioner.

### Referral statistics

The practicese referral rates between August and October 2013 and 2014 are included in table 4, whereas the referral rates between January and March 2014 and 2015 are included in table 5. The number of onward referrals made by the treating GP after being seen within the MSK and SEM clinic in the period August to October 2014 was three—two to physiotherapy and one to rheumatology—to exclude an inflammatory cause of the patient's pain, whereas the number of onward referrals from the clinic during the time period January to March 2015 was two—one to occupational therapy and one to orthopaedics—for consideration of surgical management options. This is compared with the 83 referrals which would have been made from the practice if this MSK and SEM clinic did not exist. Comparing referral rates between August and October 2013 and the same time period in 2014, overall referrals from the practice were reduced by 147, orthopaedic referrals were reduced by 2, while rheumatology referrals were reduced by 3, MSK presentations to the practice were reduced by 60, and physiotherapy and X-ray referrals were reduced by 47 and 90, respectively.

**Table 4:** Referral rates for the practice: August to October 2013 and 2014

Practice statistics	Overall practic referral rates	Orthopaedic referrals	MSK presentations within the practice	Physiotherapy referrals	X-ray referrals	Rheumatology referrals
August to October 2013	881	55	317	133	319	13
August to October 2014	732	53	257	86	229	10

*Previously published (6).

MSK, musculoskeletal.

**Table 5:** Referral rates for the practice: January to March 2014 and 2015

Practice statistics	Overall outpatient practice referral rates	Orthopaedic referrals	Overall community referrals (includes physiotherapy)	Physiotherapy referrals	X-ray referrals	Rheumatology referrals
January to March 2014	971	61	233	93	324	16
January to March 2015	819	52	159	52	321	12

Comparing the referral rates between January and March 2014 and the same time period in 2015, overall referrals to outpatients from the practice were reduced by 152, orthopaedic referrals were reduced by 9, while rheumatology referrals were reduced by 4, and physiotherapy and X-ray referral rates were reduced by 41 and 3, respectively.

### Economic evaluation

All the patients referred to the GP-based MSK clinic were seen within 4 weeks. A review of the 2013 orthopaedic ICAT waiting times in NI showed that 5833 patients (49.5%) were seen between 0 and 6 weeks of referral, 2304 patients (19.6%) between 6 and 9 weeks, 1872 patients (15.9%) waiting between 9 and 12 weeks, 1389 patients (11.8%) waiting between 12 and 15 weeks, 236 patients (2%) waiting between 15 and 18 weeks and 144 patients (1.2%) waiting more than 18 weeks to be seen (from a total of 11 778 patients). The cost in NI of a routine hospital orthopaedic outpatient review in 2014 was £213 and the average orthopaedic ICAT cost per attendance was £82 (information obtained through direct communications with the finance department of the Department of Health, Social Services and Public Safety (DHSSPS; NI)), whereas the cost of 1 h of GP-patient contact, including direct care staff costs with qualification costs, for 2013–2014 was £183 (information obtained through direct communications with the finance department of DHSSPS (NI)). Three patients were at least seen per hour and therefore the cost per patient reviewed at the GP-based MSK and SEM clinic was conservatively costed at £61 per patient in 2014. Therefore, if all the clinic's patients were reviewed within a hospital orthopaedic outpatient clinic, the cost to the NI health service would have been £17 679, or within the orthopaedic ICAT system the cost would have been £6806. This is compared with the £5063 which it cost to run the GP-based MSK and SEM clinic, a potential saving of between £1743 and £12 616 per 83 patients reviewed.

## Discussion

Thirty-five and then 48 patients were reviewed at the GP-based MSK and SEM clinic in 2014 and 2015, respectively, all within 4 weeks of initial presentation to their own GP. Patient satisfaction with the service was generally very high, with all patients preferring to be reviewed within their own GP surgery rather than being referred to a hospital or another community-based health centre, in keeping with the principles of Transforming Your Care.^5^ This study extends the analysis of the implementation of a unique MSK/SEM into routine GP to 6 months and builds on our previously published quality improvement work.^6^

### Referral trends

Onward orthopaedic referrals from the GP practice were reduced by 11 during the study period compared with the same time periods in 2014 and 2015. MSK presentations to the GP practice in 2014 were reduced by 114 compared with the same time frame in 2013. This statistic was unfortunately not available for the 2015 cohort of patients due to changes within the computer system. This reduction in MSK presentations to the GP surgery could be explained by a more efficient management of these symptoms within the surgery, with patients utilising the GP-based MSK and SEM clinic and receiving appropriate management rather than continually re-presenting to their own GP on multiple occasions. The reduction in X-ray referrals, 93 during the full study period, has potentially significant consequences for patients, with radiation from X-rays being associated with certain adverse health consequences.^9^ Five referrals were made from the clinic to secondary care (two to physiotherapy and one each to rheumatology, orthopaedics and occupational therapy). However, if this clinic did not exist, then all 83 patients seen in the clinic would have been referred to secondary care for input. This clinic therefore successfully reduced the burden on secondary care for orthopaedic and MSK referrals.

### Prevalence of various MSK conditions

The main presenting conditions/symptoms in this project related to the shoulder, with supraspinatus and subacromial bursitis being the most common area for pathology. However, the back and knee have been reported as the most common body regions causing patients to attend their GP in patients with MSK symptoms,^2^ whereas other studies concluded that the back and neck were the most common presenting areas.^3^

This difference may be explained by the fact that the GP-based MSK and SEM clinic was receiving referrals from primary care, and the GPs were therefore filtering out these other MSK presentations within their own clinics. Previous authors have also found that women present more commonly than men for MSK problems,^2^ in keeping with our findings. This information should also be utilised when teaching GPs about primary care MSK medicine and the common joints which present to GPs. This would allow GPs to feel more confident in managing common MSK and SEM symptoms and therefore reduce the secondary care referral burden.

### Economic considerations

Having a GP-based MSK and SEM clinic has the potential for significant cost savings for the NHS. Managing patients within their own GP practice through utilisation of a GP with a specialist interest in MSK and SEM conditions in this current project had a potential cost saving of between £1743 and £12 616 per 83 patients reviewed. This does not include the reductions in hospital, physiotherapy and X-ray referrals seen within this study as well as fewer MSK presentations to the GP surgery and these cost savings are therefore conservative. This economic saving was achieved with high patient satisfaction and occurs at a time when the Belfast Trust is under significant economic pressure.^10^

### Challenges, lessons and future directions

One of the main issues encountered during the process for the GP leading the MSK and SEM clinic was that the Trust does not have guidelines for patients on oral anticoagulants receiving intra-articular injections. This should be remedied by the Trust, particularly with the advent of the newer oral anticoagulant agents, although previous authors have suggested that joint injections with a therapeutic international normalised ratio is safe.^11^ ^12^

With regard to the future development of this service, it is hoped that this model will provide the blueprint for a further roll-out of GP-based MSK and SEM clinics within NI. To enable patients to receive the best possible care within these clinics, it is hoped that GPs with a specialist interest in MSK and SEM will be able to be trained in the use of MSK ultrasound—the ‘stethoscope’ of modern medicine.^13^ This skill would be able to be utilised as both an investigation and to help with patient management via, for example, ultrasound-guided injections, and the provision of this service should be reviewed to illustrate if it provides a further cost saving to the MSK and SEM service.

A further improvement to the service could be made by providing both platelet-rich plasma (PRP) and whole-blood injections in addition to the current provision of corticosteroid injections, for muscle,^14^ tendinopathic^15^ and arthritic conditions,^16–18^ if supported by an appropriate evidence base.^19^ This project also provides GPs with information on which MSK and SEM conditions commonly present to GP and which some GPs may have difficulty managing, particularly around the shoulder and knee. GPs and GP trainees should therefore be provided with appropriate education to help address this learning need and further reduce the onward referral from primary care of MSK and SEM conditions.

## Strengths and weaknesses

The review of referral rates from the GP practice is dependent on the primary care staff coding appropriately and our statistics are therefore limited by the quality of the information which the staff enters into the system. This may therefore lead to underestimating or overestimating referral rates. The statistics available from the primary care computer system, EMIS, for the two study time frames were different due to mandatory changes to the computer system. The diagnoses made were largely clinical and we would have ideally confirmed these with appropriate imaging, but a pragmatic approach was taken to investigate and manage the patients, in keeping with ‘normal’ primary care.

This study was performed in one GP practice, which allowed us 100% follow-up of our patients, but our sample size was relatively small with no long-term follow-up. The next stage for the quality improvement project will be to replicate these findings within a larger geographical area, with long-term follow-up of patients, and then present the findings to local commissioners to further extend this trial across different areas of the UK.

## Summary

With the financial constraints now faced by the NHS and new healthcare policies shifting focus from hospital-based to community-based management options, we present a novel service model for managing MSK and SEM problems in primary care. This model can make sound economic sense and deliver high patient satisfaction within primary care, with low waiting times, helping to reduce the referral burden on secondary care from primary care. We present a reproducible model that can be commissioned as a service by the local clinical commissioning groups and be extended throughout the NI and UK health service as part of Transforming Your Care policy.^5^

## Supplementary Material

Supplementary Data
