# Supplementary material for: A ‘novel’ model for integrating Sport and Exercise Medicine (SEM) and Musculoskeletal (MSK) management into primary care in the UK
Source: BMJ Open Sport Exerc Med. 2015 Sep 15;1(1):e000027. doi: 10.1136/bmjsem-2015-000027 (PMC5117016; doi:10.1136/bmjsem-2015-000027)
Supplement: Supplementary Data [file supp_1_1_e000027__index.html]

Supplementary Data -- -- BMJ Open Sport & Exercise Medicine 

# A ‘novel’ model for integrating Sport and Exercise Medicine (SEM) and Musculoskeletal (MSK) management into primary care in the UK

## Supplementary Data

This web only file has been produced by the BMJ Publishing Group from an electronic file supplied by the author(s) and has not been edited for content.

- Data supplement 1 - Online supplement
