# Supplementary material for: A ‘novel’ model for integrating Sport and Exercise Medicine (SEM) and Musculoskeletal (MSK) management into primary care in the UK
Source: BMJ Open Sport Exerc Med. 2015 Sep 15;1(1):e000027. doi: 10.1136/bmjsem-2015-000027 (PMC5117016; doi:10.1136/bmjsem-2015-000027)
Supplement: Supplementary Data [file supp_1.1.e000027_bmjsem-2015-000027supp.pdf]

## Appendix 1

## Patient Satisfaction Questionnaire

Adapted from Patient satisfaction questionnaire for general practitioner services (7).

Male ________ Female________

Age (years)____________

| | Strongly agree (1) | Agree (2) | Neither agree nor disagree (3) | Disagree (4) | Strongly disagree (5) |
|---|---|---|---|---|---|
| 1) Getting an appointment for the GP-based orthopaedic clinic at a convenient time was easy? | | | | | |
| 2) The doctor did enough tests to find out what was wrong with me? | | | | | |
| 3) I have absolute faith and confidence in the doctor at the GP-based orthopaedic clinic? | | | | | |
| 4) The doctor at the GP-based orthopaedic clinic did not tell me enough about the treatment? | | | | | |
| 5) The doctor at the GP-based orthopaedic clinic fully explained how the illness and treatment would affect my future health? | | | | | |
| 6) Appointments are easy to make whenever I need them at the GP-based orthopaedic clinic? | | | | | |
| 7) I felt perfectly satisfied with the way I was treated at the surgery when I attended the GP-based orthopaedic clinic? | | | | | |
| 8) The doctor showed a genuine interest in my problems at the GP-based orthopaedic clinic? | | | | | |

| | | | | | |
|---|---|---|---|---|---|
| 9) The doctor always puts me at ease at the GP-based orthopaedic clinic? | | | | | |
| 10) My general experience at the GP-based orthopaedic clinic was very good? | | | | | |
| 11) My experience at the GP-based orthopaedic clinic was generally better than if I had been referred to hospital for an orthopaedic outpatient review? | | | | | |
| 12) If I was referred to see a specialist, I would prefer to see him/her in my own GP practice? | | | | | |

Please feel free to add any general comments:

## Appendix 2

A forty-nine year old male wrote:

> "(the doctor and clinic are) a great asset to the surgery."

Another forty-nine year old male wrote:

> "(the reviewing doctor) made me feel at ease and talked to me like a friend. I would not hesitate to attend his clinic again."

A fifty-five year old female also commented:

"Excellent service."
